# Revisit the Correlation between the Elastic Mechanics and Fusion of Lipid Membranes

**DOI:** 10.1038/srep31470

**Published:** 2016-08-18

**Authors:** Zih-An Fan, Kuan-Yu Tsang, Si-Han Chen, Yi-Fan Chen

**Affiliations:** 1 Department of Chemical and Materials Engineering, National Central University, No. 300, Zhongda Rd., Zhongli District, Taoyuan 32001, Taiwan

## Abstract

Membrane fusion is a vital process in key cellular events. The fusion capability of a membrane depends on its elastic properties and varies with its lipid composition. It is believed that as the composition varies, the consequent change in *C*_*0*_ (monolayer spontaneous curvature) is the major factor dictating fusion, owing to the associated variation in *G*_*E*_s (elastic energies) of the fusion intermediates (e.g. stalk). By exploring the correlations among fusion, *C*_*0*_ and *K*_*cp*_ (monolayer bending modulus), we revisit this long-held belief and re-examine the fusogenic contributions of some relevant factors. We observe that not only *C*_*0*_ but also *K*_*cp*_ variations affect fusion, with depression in *K*_*cp*_ leading to suppression in fusion. Variations in *G*_*E*_s and inter-membrane interactions cannot account for the *K*_*cp*_-fusion correlation; fusion is suppressed even as the *G*_*E*_s decrease with *K*_*cp*_, indicating the presence of factor(s) with fusogenic importance overtaking that of G_*E*_. Furthermore, analyses find that the *C*_*0*_ influence on fusion is effected via modulating *G*_*E*_ of the pre-fusion planar membrane, rather than stalk. The results support a recent proposition calling for a paradigm shift from the conventional view of fusion and may reshape our understanding to the roles of fusogenic proteins in regulating cellular fusion machineries.

Membrane fusion is vital for living organisms. Many cellular events, such as the release of neurotransmitters, the invasion of enveloped viruses, the intracellular trafficking of proteins and the conception for sexual reproduction, involve membrane fusion^1^,^2^. Complete of the fusion process sees two membrane-bound entities merge into a single one, with the initially discrete membranes and the enclosed contents mixed together. Cellular implementation of fusion requires the concerted action of an intricate machinery consisting of lipids, fusogenic proteins and fusion-triggering stimulants (*e*.*g*., Ca^2+^)^3^,^4^. While the wide diversity of the lipids, proteins and other biomolecules involved in cellular fusion often complicates the attempts to explore the inner working shared by various fusion machineries, protein-free model membranes with defined lipid compositions [*e*.*g*., liposome, also known as unilamellar vesicle (ULV), a hollow spherical structure bound with a *single* lipid bilayer] have been proven an indispensable tool in uncovering the universal mechanism for all sorts of fusion^3^,^5^.

It is known from model membrane studies that initiating and advancing the fusion process demand the overcoming of several energy barriers; recognizing these barriers has provided insight on how proteins regulate cellular fusion machineries^3^,^5^. The first energy barrier arises from the need to bring two fusion-destined membranes into close proximity to initiate fusion^6^,^7^. The barrier, an inter-membrane interaction known as hydration repulsion, results from the resistance to removing inter-membrane water needed for shortening the inter-membrane distance^8^. Once fusion is initiated, the next energy barriers are related to the structural transformations of the *cis*-monolayers (the proximal leaflets of the lipid bilayers undergoing fusion)^4^,^9^. Theoretical studies predict that the monolayers transform from the initial planar conformation to two fusion intermediate structures, hemifusion stalk and hemifusion diaphragm (HD), before forming a fusion pore (FP) to complete the process. The hemifusion structures are highly curved. Analogous to the well-studied lamellar ↔ nonlamellar phase transitions of lipid dispersions^10^, the transition from planar monolayers to the hemifusion structures also entails the membrane deformations that implicate the *monolayer* elastic energy density, *g*_*E*_,


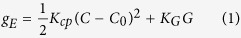


where *K*_*cp*_ is the monolayer bending modulus, *C* the total curvature, *C*_*0*_ the monolayer spontaneous curvature (the molar-weighted average of the *C*_*0*_s of the constituting lipids), *K*_*G*_ the Gaussian modulus and *G* the Gaussian curvature^11^,^12^. When free of any external constraint, a monolayer of a specific *C*_*0*_ tends to form a structure with *C* = *C*_*0*_. Any deviation of *C* from *C*_*0*_ increases *g*_*E*_, destabilizes the monolayer structure possessing the given *C* and raises the energy barrier to the formation of this structure. Accordingly, a monolayer having |*C*_*0*_| >> 0 exhibits a high tendency of forming nonlamellar structures, with the sign of the *C*_*0*_ indicating its preference for normal (positive) or inverted (negative) nonlamellar structures, while monolayers with |*C*_*0*_| ≈ 0 prefer a planar conformation.

The energy barriers, and thus the fusion capability, can be reduced/raised by tuning the membrane composition. Experiments show that adjusting the composition of a ULV alters its fusion capability considerably, which is attributable to the consequent changes in *g*_*E*_ and/or hydration repulsion^6^,^13^. Conventionally, the former is considered as the major factor affecting fusion and is induced by introducing to membranes the lipids of negative *C*_*0*_s, because hemifusion stalk, whose existence has been experimentally confirmed^14^, is a structure with negative *C*s for the *cis*-monolayers (Fig. 1). Nevertheless, varying *C*_*0*_ is merely one approach to change *g*_*E*_; varying *K*_*cp*_ can be another, as manifested in Equation (1). While many efforts have been dedicated to exploring the effects of varying *C*_*0*_ on fusion and their underlying mechanisms, few, if any, take on the issue of how, or even whether, the change in *K*_*cp*_ affects the energy barriers and thus fusion of a membrane, even though such investigations will surely provide further insight. Moreover, a recent X-ray study demonstrated the need to shift the conventional “*C*_*0*_-centered” view on fusion because hydration repulsion was proven as energetically important as *g*_*E*_, if not more so, to fusion^6^. It is therefore of great value to re-examine the fusogenic importance of elastic mechanics and explore the fusogenic relevance of other factors.

This study aspires to address these issues and provide further insight on the fusion mechanisms of protein-free lipid membranes, in the hope that the learned knowledge will advance our understanding to the working principles general to fusogenic proteins. Several experimental techniques (*e*.*g*., X-ray diffraction, the electron density reconstruction and fluorescence spectroscopy) have been employed and an experimental scheme (specifically for the *K*_*cp*_ measurements) developed to measure 1) the *C*_*0*_s and *K*_*cp*_s of several lipid species [*i*.*e*., the species carrying 18 carbon-long tails of various saturation degrees: dioleoylphosphatidylethanolamine (DOPE or 18:1 PE), 18:2 PE, 18:3 PE, dioleoylphosphatidylcholine (DOPC or 18:1 PC), 18:2 PC and 18:3 PC] and 2) the fusion efficiencies of ~150-nm ULVs (*i*.*e*., large unilamellar vesicles, LUVs) as functions of *C*_*0*_ and *K*_*cp*_ via tuning the lipid composition. To the best of our knowledge, the measurements are the first systematic investigation on the correlations among *C*_*0*_, *K*_*cp*_, the hydrocarbon (CH) chain saturation of a lipid and fusion. Energetic analyses on the *C*_*0*_- and *K*_*cp*_-dependences of fusion reveal the fusogenic relevance of a factor(s) other than elastic mechanics and inter-membrane interactions, whose fusogenic importance even overtakes that of *C*_*0*_ and *G*_*E*_, and also provide a new energetic perspective on how fusion is promoted by making *C*_*0*_ more negative. The results complement the earlier studies by expanding the known effects of changing the lipid composition on the fusion energetics, and may reshape the current understanding to the fusion mechanism.

## Results

### Elastic properties of DOPE and DOPC

The *C*_*0*_ and *K*_*cp*_ of DOPE are measured to compare against the published data to establish credibility for our experimental protocol and data. The *C*_*0*_ of DOPE measured here, *C*_*0*,*DOPE*_ = −0.0334 ± 0.0001 Å^−1^ [=1/(−29.9 ± 0.1) Å^−1^] at 20 °C (Table 1), agrees well with the literature [*e*.*g*., *C*_*0*,*DOPE*_ = 1/(−30.2 ± 0.8) Å^−1^ at 20 °C; *C*_*0*,*DOPE*_ = 1/(−30) Å^−1^ at 20 °C]^15^,^16^. The thermal variation rate of *C*_*0*,*DOPE*_, −0.00014 ± 0.00004 Å^−1^ °C^−1^, is also consistent with the reports by refs 15,17 (−0.00017 Å^−1^ °C^−1^ and −0.00013 ± 0.00004 Å^−1^ °C^−1^, respectively). A similar consistency is also observed for *K*_*cp*_, where *K*_*cp*,*DOPE*_ = 0.57 ± 0.12 × 10^−19^ J (≈13.9 ± 2.9 K_B_T) at 25 °C conforms to, *e*.*g*., *K*_*cp*,*DOPE*_ = 0.46 × 10^−19^ J (≈11.2 K_B_T) at 20 °C in ref. 16 and *K*_*cp*,*DOPE*_ = 0.53 × 10^−19^ J (≈12.9 K_B_T) at 22 °C in ref. 18.

The *C*_*0*_ of DOPC, derived from the linear correlation between *C*_*0*_ and lipid composition (see Methods), is measured as *C*_*0*,*DOPC*_ = −0.0111 ± 0.001 Å^−1^ [=1/(−90.1 ± 8.1) Å^−1^] at 30 °C and *C*_*0*,*DOPC*_ = −0.0116 ± 0.0009 Å^−1^ [=1/(−86.2 ± 6.7) Å^−1^] at 35 °C. These are comparable to *C*_*0*,*DOPC*_ = 1/(−110.0 ± 9.7) Å^−1^ at 35 °C in ref. 17 and *C*_*0*,*DOPC*_ = 1/(−87.3) Å^−1^ at 32 °C in ref. 18. (Note that the uncertainties for *C*_*0*,*DOPC*_s in this study and ref. 17 are intrinsic to the *C*_*0*_-determination method and do not arise from experimental errors.) The thermal variation rate of *C*_*0*,*DOPC*_, −0.00010 ± 0.00002 Å^−1^ °C^−1^, is also agreeable with −0.00011 ± 0.00006 Å^−1^ °C^−1^ in ref. 17. The consistency with the published data validates the protocol used here and establishes credibility for the data below.

### Elastic properties of the polyunsaturated PEs and PCs

**Figure 2:**
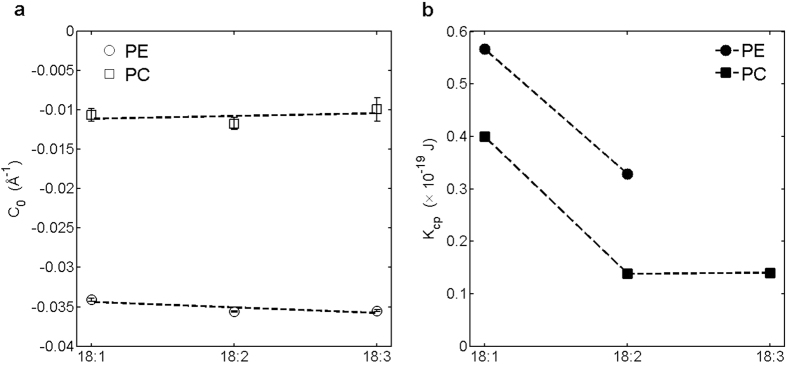
Dependences on the CH chain saturation of (**a**) the *C*_*0*_s and (**b**) the *K*_*cp*_s of PE and PC. Note the K_cp,DOPC_ is adopted from refs 17,18.

The *C*_*0*_ measurements for the unsaturated PEs and PCs show that carrying tails with different saturation degrees has only marginal effects on the *C*_*0*_s of the lipids, with the *C*_*0*_s barely shifting from *C*_*0*,*PE*_ =  −0.0341 ± 0.0002 Å^−1^ [=1/(−29.3 ± 0.2) Å^−1^] to −0.0355 ± 0.0001 Å^−1^ [=1/(−28.2 ± 0.1) Å^−1^] and from *C*_*0*,*PC*_ = −0.0106 ± 0.0008 Å^−1^ [=1/(−94.3 ± 7.1) Å^−1^] to −0.0099 ± 0.0015 Å^−1^ [=1/(−101.0 ± 15.3) Å^−1^] when the CH chains go from monounsaturated to triunsaturated at 25 °C (Fig. 2a). This is unexpected, since CH chains with higher unsaturation degrees are more flexible and shall be more capable of assuming the splaying-out conformations, making the lipid molecules more cone-shaped and the *C*_*0*_s more negative, as in raising temperature.

On the contrary, *K*_*cp*_ is dependent on the saturation degree. The *K*_*cp*_s of PE and PC are smaller for the lipids carrying tails with higher unsaturation degrees (Fig. 2b). A monolayer is thus softer when constituted by di- or triunsaturated lipids than would be when constituted by the monounsaturated counterparts. Together with *K*_*cp*,*DOPC*_ = 0.42 ± 0.05 × 10^−19^ J at 18 °C from ref. 19 and *K*_*cp*,*DOPC*_ = 0.40 ± 0.04 × 10^−19^ J at 30 °C from ref. 20, *K*_*cp*,*PC*_ varies from *K*_*cp*,*PC*_ = ~0.4 × 10^−19^ J (≈9.7 K_B_T) to 0.14 × 10^−19^ J (≈3.4 K_B_T) when the number of the *cis*-double bonds in a CH chain goes from 1 to 3 (Fig. 2b). A similar trend is observed for PE: *K*_*cp*,*PE*_ decreases from *K*_*cp*,*PE*_ = 0.57 × 10^−19^ J (≈13.9 K_B_T) to 0.33 × 10^−19^ J (≈8 K_B_T) when the chain saturation goes from monounsaturated to diunsaturated. Overall, variations by a factor of ~2 are observed for PE and PC when the CH chains go from monounsaturated to polyunsaturated.

It is noted that our results for *K*_*cp*,*PC*_ are consistent with ref. 19: The *K*_*cp*,*PC*_ in the reference (measured with the micropipette-aspiration technique) declined sharply from *K*_*cp*,*PC*_ = 0.42 ± 0.05 × 10^−19^ J (≈10.2 ± 1.2 K_B_T) to 0.22 ± 0.04 × 10^−19^ J (≈5.3 ± 1 K_B_T) as the chains go from monounsaturated to diunsaturated, but the *K_cp,PC_* changed only modestly when the chains go further to triunsaturated. (Note that the property measured in ref. 19 was the *bilayer* bending modulus. Following ref. 21, this is converted to its monolayer counterpart by dividing it by 2, with the assumption of no coupling between the two monolayers). Even with different experimental techniques and an oversimplified assumption of no inter-monolayer coupling, the discrepancy between the *K*_*cp*_s obtained here and in ref. 19 is still remarkably small. The consistency warrants the application of the experimental scheme developed here (see Methods) to measuring the *K*_*cp*_s of the lamellar-forming lipids.

### *C*_*0*_ and *K*_*cp*_ dependences of fusion

Based on the *C*_*0*_ and *K*_*cp*_ measurements, we manage to tune the composition of a monolayer so that one of the two properties varies while the other remains constant. This objective is achieved satisfyingly for varying *K*_*cp*_ but only with limited success for *C*_*0*_. With such a composition control, we study the correlation between fusion and *K*_*cp*_ (or *C*_*0*_) for LUVs. Two series of LUVs are prepared: one series composed of DOPE and DOPC with the molar fraction varied; the other composed of equimolar DOPE and PC with the PC selected from among DOPC, 18:2 PC or 18:3 PC. Fusion is initiated by PEG 8000 and detected/quantified fluorescently (fluorescence is emitted after fusion due to the complexation of the dyes, DPA and TbCl_3_, initially encapsulated in separate LUVs; see Methods)^22^.

**Figure 3:**
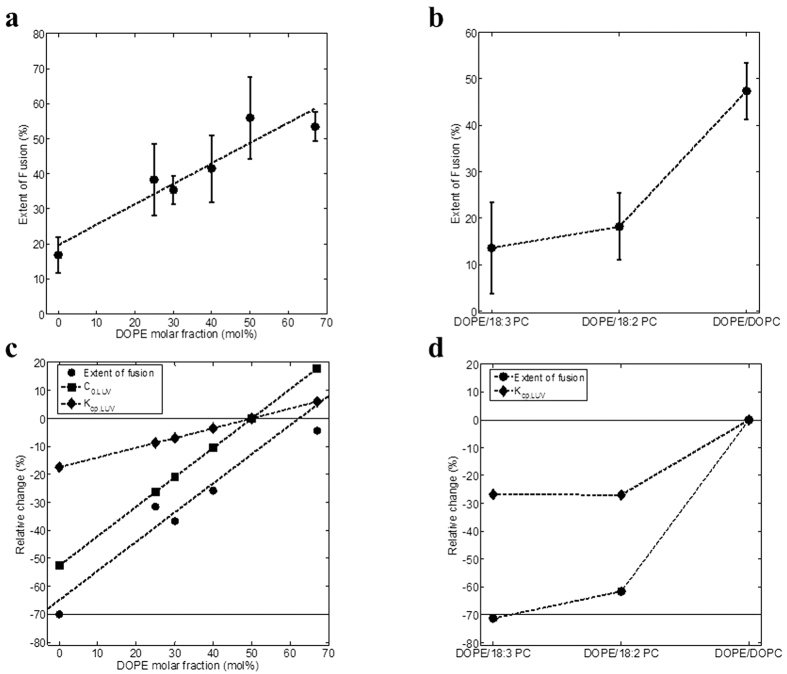
Fusion behavior for (**a**) the DOPE/DOPC and (**b**) the equimolar DOPE/PC LUVs. The relative changes in fusion, *C*_*0*,*LUV*_ and *K*_*cp*,*LUV*_ as functions of (**c**) DOPE fraction and (**d**) PC chain saturation display the correlation among fusion, *C*_*0*_ and *K*_*cp*_. The dashed lines are to guide the eyes; the solid lines in (**c**,**d**) mark the 0% and 70% changes.

The *C*_*0*_ dependence of fusion is studied by examining fusion between the DOPE/DOPC LUVs. The *monolayer* spontaneous curvature of the LUVs, *C*_*0*,*LUV*_, varies from −0.0106 ± 0.0008 Å^−1^ [=1/(−94.3 ± 7.1) Å^−1^)] to −0.0264 ± 0.0003 Å^−1^ [=1/(−37.9 ± 0.4) Å^−1^], while the *monolayer* bending modulus, *K*_*cp*,*LUV*_ (assumed to be the molar-weighted average of *K*_*cp*,*DOPE*_ and *K*_*cp*,*DOPC*_), is kept at a narrower range of 0.4 × 10^−19^ J (≈9.7 K_B_T) to 0.51 × 10^−19^ J (≈12.2 K_B_T), when DOPE rises from 0 mol% to 67 mol% (Table 1). As the DOPE fraction rises, the extent of fusion for the LUVs also rises (Fig. 3a). A comparison where the relative changes [relative change = 100% × {(property determined at a given [DOPE]) – (property determined at [DOPE] = 50 mol%)}/{property determined at [DOPE] = 50 mol%}] in *C*_*0*_, *K*_*cp*_ and fusion are plotted against DOPE fraction demonstrates the expected correlation between fusion and *C*_*0*,*LUV*_ reported in the literature (Fig. 3c): It was reported that ULVs with higher contents in lipids of negative *C*_*0*_s, or prepared with lipids of more negative *C*_*0*_s, displayed a higher propensity for fusion^13^,^23^, while introducing to ULVs lipids of strongly positive *C*_*0*_s could inhibit fusion^24^, whether the systems were protein-free or contained fusogenic proteins. However, it has to be stressed that the variation in *K*_*cp*,*LUV*_ may still be substantial enough to sway fusion, which is further discussed below.

The *K*_*cp*_ dependence of fusion is studied on the DOPE/PC LUVs. Substituting 18:2 PC or 18:3 PC for DOPC in the LUVs varies the *K*_*cp*,*LUV*_ from 0.49 × 10^−19^ J (≈11.8 K_B_T) to 0.35 × 10^−19^ J (≈8.6 K_B_T) or 0.36 × 10^−19^ J (≈8.6 K_B_T) but retains the *C*_*0*,*LUV*_ at ~−0.0220 Å^−1^ [=1/(−45.5) Å^−1^] (Table 1). Surprisingly, the fluorescence intensity emitted by the dye complexes decreases drastically when the CH chains of PC go from monounsaturated to polyunsaturated; in some instances, the fluorescence for the DOPE/18:3 PC LUVs is even nearly undetectable (Fig. 3b). This sharp decline in fluorescence may originate from 1) a failure to load the dyes into the LUVs, 2) a failure to form LUVs or 3) a depression in fusion of the LUVs. The first two scenarios are excluded because (I) the fluorescence was fully recovered when the structures self-assembled by DOPE/polyunsaturated PC (whether they were LUVs or not) were disrupted by Triton X-100 (a detergent), indicating that the dyes had initially been sequestered by the self-assembled structures; and (II) the self-assembled structures were ~150 nm in size, as with the LUVs made for the rest of the study, suggesting the self-assembled structures being LUVs. Hence, we consider the reduction in fusion as the most probable cause for the sharp decline in fluorescence when the *K*_*cp*,*LUV*_ decreases, thereby establishing a correlation between fusion and *K*_*cp*,*LUV*_ (Fig. 3d).

## Discussion

### The rise in *G*_*E*_ of the planar monolayer, rather than the drop in *G*_*E*_ of stalk, is responsible for the fusion promotion induced by making *C*_*0*_ more negative

**Figure 4:**
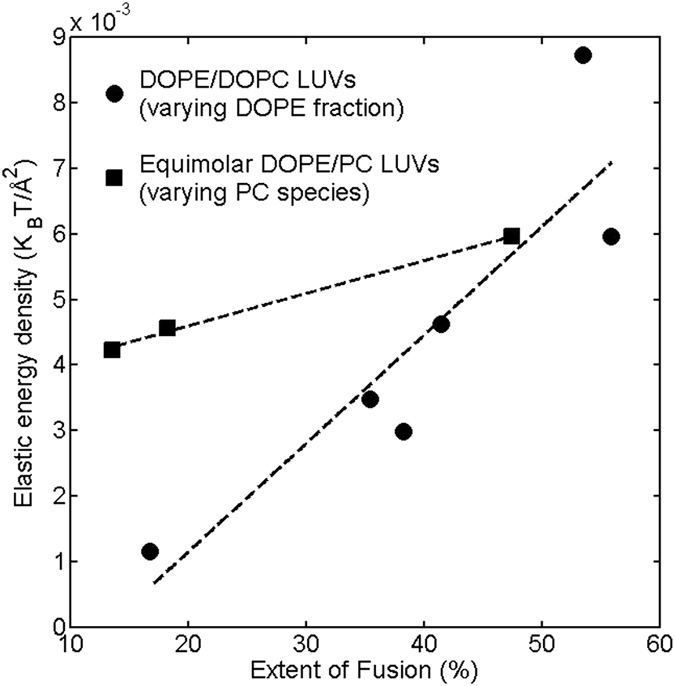
*g*_*E*,*LUV*_ versus fusion for the DOPE/DOPC LUVs with the DOPE fraction varied, and for the equimolar DOPE/PC LUVs with the PC species changed. The dashed lines are to guide the eyes.

The fusion promotion by making *C*_*0*_ more negative has long been associated with the energetics of the fusion intermediate structures, especially hemifusion stalk^3^. Due to its hourglass shape, stalk is more energetically favorable, while a planar conformation is less favored, if the *C*_*0*_s of the underlying monolayers are more negative^25^. Indeed, experiments show that using the lipid of highly negative *C*_*0*_, diphytanoyl phosphatidylcholine (DPhPC), to prepare oriented bilayers allowed researchers to determine the first X-ray diffraction structure for stalk^14^; and the difference between the curvature energies [the first term in Equation (1)] of stalk and planar monolayers dropped triply when the composition of the oriented bilayers changed from DOPC alone to equimolar DOPE/DOPC, contributing to the lowering of the osmotic stress minimally needed for stabilizing stalk^6^. However, it has not been explicitly verified from experimental data whether the preference for stalk is mainly due to a rise in the elastic energy of planar monolayers or a drop in that of stalk, even though the latter is implicitly assumed to be the case in many studies. To resolve the question, we calculate the elastic energy density of a LUV, *g*_*E*,*LUV*_, for the DOPE/DOPC LUVs via Equation (1). With the measured diameter of ~150 nm (see Methods) and the common bilayer thickness of ~4 nm for the LUVs, the *C*s of the inner and outer leaflets are −(1/740 + 1/740) Å^−1^ and + (1/760 + 1/760) Å^−1^, respectively, while the Gaussian curvatures (1/740 × 1/740 Å^−2^ and 1/760 × 1/760 Å^−2^) are negligible. (With the dimensions, the bilayer can be regarded as locally flat). Based on this and the *C*_*0*_/*K*_*cp*_ data, *g*_*E*,*LUV*_* = g*_*E*,*inner leaflet*_ + *g*_*E*,*outer leaflet*_ is determined (Table 2). The *g*_*E*,*LUV*_ steadily rises along with the DOPE fraction, leading to its positive correlation with fusion (the Pearson correlation coefficient is 0.8889, Fig. 4), *i*.*e*., the higher the *g*_*E*,*LUV*_ is, the more the LUVs are inclined to fusing. The variation in *C*_*0*_ alone accounts for >75% of the change in *g*_*E*,*LUV*_. Thus, the result establishes a correlation among *C*_*0*_, *g*_*E*,*LUV*_ and fusion: Making *C*_*0*_ more negative elevates *g*_*E*,*LUV*_, which in turn promotes fusion. More specifically, making *C*_*0*_ more negative aggravates the energetic penalty of keeping the monolayers in the planar conformation, causing the transformation to stalk more appealing in comparison.

The remaining question is: Does making *C*_*0*_ more negative also reduce the elastic energy of stalk? We deduce the elastic energies (defined against the reference point, the elastic energy of a monolayer with *C*_*0*_ = 0 and in the planar conformation), *G*_*E*_s, of the *cis*-monolayers in the planar and stalk conformations. The difference, Δ*G*_*E*,*ps*_, between *G*_*E*_s of the two conformations can be expressed as^6^,


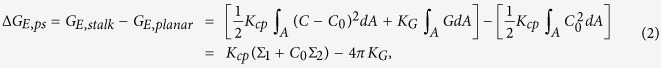


with


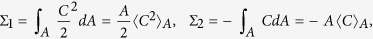


(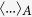 is the average over surface area *A*) and,


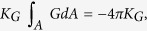


for stalk. Based on our *C*_*0*_/*K*_*cp*_/*g*_*E*,*LUV*_ data, (Σ_1_, Σ_2_) = (7.84, 207.6) for DOPC alone and (Σ_1_, Σ_2_) = (6.81, 200.5) for equimolar DOPE/DOPC^6^ and *K*_*G*_ ≈ 8 K_B_T^26^, Δ*G*_*E*,*ps*_ is determined to be ~155 K_B_T and ~128 K_B_T for the *cis*-monolayers made of DOPC alone and equimolar DOPE/DOPC, respectively. The shrink in the Δ*G*_*E*,*ps*_ upon introducing DOPE is consistent with what is seen for *g*_*E*,*LUV*_. By adopting from ref. 6 the surface areas of ~7,600 Å^2^ for monolayers made of DOPC alone and ~8,700 Å^2^ for equimolar DOPE/DOPC, *G*_*E*_ of the *cis*-monolayers in the planar conformation is determined as *G*_*E*,*planar*_ = *g*_*E*,*outer leaflet*_ × 7,600 = 6.5 K_B_T (or 0.27 × 10^−19^ J) for DOPC alone and 32.0 K_B_T (or 1.32 × 10^−19^ J) for equimolar DOPE/DOPC. Again, the result is consistent with the observation for *g*_*E*,*LUV*_. We deduce *G*_*E*_ of stalk by summing the corresponding Δ*G*_*E*,*ps*_ and *G*_*E*,*planar*_. Strikingly, the deduced *G*_*E*,*stalk*_ is ~160 K_B_T for the *cis*-monolayers made of both DOPC alone and equimolar DOPE/DOPC. In contrast, *G*_*E*,*planar*_ differs by a factor of 5 or ~26 K_B_T upon the same change in the composition.

The invariability of *G*_*E*,*stalk*_ may suggest that *G*_*E*,*stalk*_ is a conserved property for monolayers and irrespective of the composition, at least for the two compositions considered here. Given the intimacy between *G*_*E*_ and the overall geometry of a structure, this may further imply that the overall geometry of stalk is also universal among monolayers of various compositions. Indeed, experimental and computational studies have shown that the overall geometry of stalk is highly conserved among monolayers of diverse compositions^6^, as well as among various coarse-grained models used in the computational studies^9^. Given the energetic/geometric invariability of stalk and the variation in *G*_*E*,*planar*_ upon changing *C*_*0*_, we argue that the fusion promotion by making *C*_*0*_ more negative is effected mainly via a rise in *G*_*E*,*planar*_, rather than a drop in *G*_*E*,*stalk*_.

Some may wonder: Equation (1) demands *g*_*E*,*stalk*_ to vary with *C*_*0*_ if *C* is constant (as seemingly entailed by the geometric invariability of stalk). Thus, how can *G*_*E*,*stalk*_ remains unchanged when the composition varies? One has to realize that the conservation in geometry is *not* equivalent of the conservation in *dimension*, or more specifically, in *C. C* of stalk may vary with the composition so that *G*_*E*,*stalk*_ is unchanged. Indeed, the X-ray diffraction structures of stalk indicate that 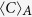 of stalk is ~1/36.6 Å^−1^ for DOPC alone and ~1/43.4 Å^−1^ for equimolar DOPE/DOPC^6^.

### Changes in the inter-membrane interactions and in *G*_*E*_s of stalk, HD and FP cannot account for the fusion reduction upon raising the chain unsaturation

Several factors may account for the apparent *K*_*cp*,*LUV*_-fusion correlation (Fig. 3d). Among these factors is the modulation of the inter-membrane repulsion arising from the membrane undulational motion (*i*.*e*., the out-of-plane fluctuation of membrane); enhancing the repulsion would prevent the LUVs from shortening the inter-membrane distances and thus stymie the fusion initiation. The undulation repulsion is entropic in nature, and proportional to temperature while inversely proportional to *K*_*cp*_^27^; the inverse proportionality to *K*_*cp*_ is consistent with the observed *K*_*cp*,*LUV*_-fusion correlation. To explore the fusogenic relevance of the undulation repulsion, we examine the sizes of the equimolar DOPE/PC LUVs before and after the addition of PEG 8000 with dynamic light scattering. Interestingly, while the DOPE/DOPC LUVs increase their diameters from ~150 nm to ~600 nm, as expected for fusion, the diameter of DOPE/18:2 PC LUVs also expands considerably from ~150 nm to >600 nm (Supplementary Fig. S5). Together with the result of the content-mixing fusion assays for the latter LUV species (Fig. 3b), we infer that raising the chain unsaturation does not prevent the LUVs from approaching one another but still considerably compromises their capabilities of completing the fusion process. Therefore, enhancement of the inter-membrane repulsions is not expected to be responsible for the correlation between chain saturation and fusion.

The apparent *K*_*cp*,*LUV*_-fusion correlation may alternatively arise from the influence on *G*_*E*_ of varying *K*_*cp*_. We again deduce *G*_*E*,*planar*_ and Δ*G*_*E*,*ps*_ to explore the underlying mechanism in the context of stalk formation. The surface area of the *cis*-monolayers is assumed to remain ~8,700 Å^2^, even though the PC of the equimolar DOPE/PC monolayers is either 18:2 PC or 18:3 PC rather than DOPC (the interfacial areas of lipids with the same headgroup but distinct chains differ marginally when they are in the same phase at a given temperature)^28^,^29^. The deduced *G*_*E*,*planar*_s are 32.0 K_B_T, 24.4 K_B_T and 22.8 K_B_T for DOPE/DOPC, DOPE/18:2 PC and DOPE/18:3 PC, respectively. The variation is modest in comparison with the case where the DOPE fraction (and *C*_*0*_) of the DOPE/DOPC monolayers is varied. On the other hand, we expect the energetic/geometric invariability of stalk to be valid for the DOPE/PC monolayers and *G*_*E*,*stalk*_ to remain ~160 K_B_T, because the stalk geometry is highly conserved among monolayers made of a wide variety of lipids (including DPhPC, which carries bulky CH chains)^6^. Hence, Δ*G*_*E*,*ps*_ is ~136.5 K_B_T for both DOPE/18:2 PC and DOPE/18:3 PC. Thus, varying *K*_*cp*_ by ~27%, while steadying *C*_*0*_ at ~−0.0220 Å^−1^, only raises Δ*G*_*E*,*ps*_ from ~128 K_B_T to ~136.5 K_B_T. Interestingly, this modest rise by ~8.5 K_B_T is sufficient to reduce fusion by 70% (Fig. 3d), while the same extent of decline in fusion is reached only when Δ*G*_*E*,*ps*_ increases by ~27 K_B_T for the case of varying the DOPE fraction (Fig. 3c). The large discrepancy between the two cases indicates that an extra factor(s), other than the elastic energy involved in the planar-to-stalk transformation, may act to dictate fusion. Indeed, as shown in Fig. 4, the variation in fusion is less correlated with *g*_*E*,*LUV*_ for varying the PC species than for varying the DOPE fraction.

In addition to the one related to stalk, *G*_*E*_s of HD and FP may also contribute to the apparent *K*_*cp*,*LUV*_-fusion correlation. To examine the significance of these contributions, we numerically estimate how the *G*_*E*_s would vary with *K*_*cp*,*LUV*_ upon substituting 18:2 PC for DOPC through,


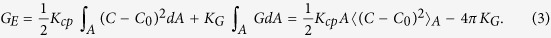


*K*_*G*_s of the LUVs are estimated by multiplying their *K*_*cp*_s with the ratio of –*K*_*G*_/*K*_*cp*_, which is about 0.84 for DOPC, DOPE and even their mixtures with other lipids (e.g., DOPC/sphingomyelin/cholesterol)^30^. Integrating *G* over a given surface area also yields −4π for both HD and FP due to the identity of their topological genus to that of stalk^31^. Due to the lack of information on how *C*s of HD and FP vary locally and with the composition, we adopt a wide range of *Cs*, −0.05 ≤ *C* ≤ + 0.018, for our estimation to include all possible variations in *C*, with the lower limit more negative than any known negative *C*_*0*_ for a phospholipid^17^ and the upper limit even greater than *C*s of the most curved region of FP^32^. The result shows that *G*_*E*,*HD*_ and *G*_*E*,*FP*_ would be constantly higher for DOPE/DOPC than for DOPE/18:2 PC, however *C* varies locally and with the composition (Table 3). This result contradicts with the observation that the DOPE/DOPC LUVs fuse more readily than the DOPE/18:2 PC LUVs (Fig. 3b), thus suggesting the irrelevance of *G*_*E*,*HD*_ and *G*_*E*,*FP*_ to the observed difference in fusion.

## Conclusion

Overall, neither the modulation of inter-membrane interactions nor the changes in Δ*G*_*E*,*ps*_, *G*_*E*,*stalk*_, *G*_*E*,*HD*_ and *G*_*E*,*FP*_ (*G*_*E*,*HD*_ and *G*_*E*,*FP*_ decrease even when fusion is suppressed!) can account for the apparent *K*_*cp*,*LUV*_-fusion correlation. This may indicate the presence of an unrecognized factor that is subject to *K*_*cp*_ and dictates fusion. It is known from Fig. 3c,d that fusion is depressed by ~70% when one either decreases *K*_*cp*_ by ~17% *plus C*_*0*_ by >50%, or simply decreases *K*_*cp*_ by ~27% without varying *C*_*0*_. It seems that depressing *K*_*cp*_ to enhance this unrecognized factor alone is sufficient to suppress fusion to a great extent; if *K*_*cp*_ is slightly less depressed, the same extent of fusion suppression is achieved only when *C*_*0*_ is simultaneously changed by a much larger degree. It is thus tempting to claim that this factor is at least as important as *C*_*0*_ and *G*_*E*_ in dictating fusion, if not more so. Another possibility is that the variation in *K*_*cp*_ is only a consequence of changing the LUV composition and does not directly affects fusion; it is another effect arising from the composition change that is responsible for the differential fusion capabilities. One such candidate is the variation in the tilt modulus, which is associated with the CH chain stretching and tilting when the chains are arranged to form stalk and HD^33^,^34^. Indeed, raising the chain unsaturation increases the tilt modulus^35^,^36^ and may thus aggravate the related energetic penalties of maintaining stalk and HD^34^. Another candidate is the variation in the hydrophobic interactions arising from exposing the CH chains of *cis*-monolayers to water during the stalk formation^34^. Nevertheless, quantifying the contributions of the two effects is out of the scope of the paper. Further studies are still desired to quantify the effects or to identify other responsible factor(s).

Conventionally, upon introducing to membranes lipids of highly negative or positive *C*_*0*_s, the change in fusion is often attributed to the change in *C*_*0*_ (and consequently in *G*_*E*_), while the accompanying variations in *K*_*cp*_ and/or other potential factors are overlooked. By examining the apparent *K*_*cp*,*LUV*_-fusion correlation, we have demonstrated that a factor(s) other than the variations in *C*_*0*_ and *G*_*E*_, which is affected by or accompanies the *K*_*cp*_ variation, plays a key role in dictating fusion when the membrane composition is changed; the fusogenic importance of the factor(s) may even overtake the importance of *C*_*0*_ or membrane elastic energetics in general. We therefore suggest that caution shall be used when one interprets the influence on fusion of varying *C*_*0*_ via membrane composition changes. This understanding, along with the conclusion that promoting fusion by making *C*_*0*_ more negative is effected via a rise in *G*_*E*,*planar*_, may reshape the energetic considerations on fusion, particularly the roles of fusogenic proteins in regulating cellular fusion machineries.

This study also made two intriguing observations: (1) Modulating the chain saturation (with the length fixed) of a lipid from monounsaturated to triunsaturated has virtually no effect on its *C*_*0*_. The observation falsifies the expectation that raising the unsaturation degree always makes *C*_*0*_ more negative; (2) our developed scheme enables the application of the osmotic stress method to the *K*_*cp*_ measurements for lipids not preferring the formation of the hexagonal H_II_ phase (formation of the phase is a prerequisite for the conventional osmotic stress method), providing an extra means for the *K*_*cp*_ determination.

## Methods

### *C*_*0*_ Measurement

Excessive buffer containing 10 mM HEPES with pH ≈ 7.4 was used to suspend a dried mixture of lipid and tetradecane. The dispersion was shuffled between two glass syringes for >100 runs and underwent >10 freeze-thaw cycles for homogenization. Each sample had *a* fixed lipid composition and contained 16 wt% tetradecane. The measurements by X-ray diffraction were carried out with Cu *Kα* or synchrotron radiation (BL13A1 and BL23A1 of NSRRC, Hsinchu, Taiwan). The diffraction images were recorded with a Pilatus 100 K pixel (Dectris, Switzerland), a Mar165 CCD and a Pilatus 1MF pixel detectors for Cu *Kα*, BL13A and BL23A1, respectively.

Data were collected at 15 °C to 40 °C with a 5 °C interval. The reduced, azimuthally integrated and background-subtracted 1-D diffraction profiles of the lipids forming the H_II_ phase were used to reconstruct the electron density profiles through 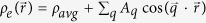 and 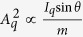, where *I*_*q*_ is the diffraction intensity, sin *θ* the Lorentz correction and *m* the multiplicity factor^37^. Following the method in ref. 15, the radial distances, *R*_*p*_s, between the H_II_ center and the pivotal plane were extracted from the profiles and experimentally determined the *C*_*0*_s of the H_II_-forming PEs. For the PCs, which preferred the formation of lamellar phases, a series of binary DOPE/PC mixtures were prepared; the molar fractions were controlled such that H_II_ was the sole stable phase in excess water and tetradecane and the mixture *C*_*0*_s were determined as for DOPE alone. The *C*_*0*_s showed linear correlations with the PC fraction (Supplementary Fig. S1). Extrapolating the correlations to pure PC yielded its *C*_*0*_. A sample, with a fixed lipid composition, only contributed to a data point in Supplementary Fig. S1.

### *K*_*cp*_ Measurement

Except that the buffer contained PEG 8000 and the data were taken at 25 °C only, the samples were prepared similarly as above. Given the Flory radius of ~8 nm, PEG molecules could not enter the H_II_ water core (<4 nm) and thus applied an osmotic stress on H_II_. The consequent structural deformation of H_II_ was given by 
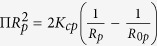
, where Π is the osmotic stress and *R*_*op*_ = 1/*C*_*0*_^16^. The slopes of the 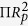-versus-1/*R*_*p*_ relation gave the *K*_*cp*_s of the H_II_-forming PEs. An experimental scheme was developed to measure the *K*_*cp*_s of the lamellar-forming PCs. A series of binary DOPE/PC mixtures were prepared and individually subjected to various osmotic stresses when H_II_ was the sole stable phase in excessive buffer and tetradecane. Supplementary Fig. S2 exemplifies the data thereby collected. Based on such data, we obtained the 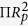-versus-1/*R*_*p*_ relations for DOPE/18:3 PC and DOPE/18:2 PC of various molar ratios (Supplementary Fig. S2b) and extracted the *K*_*cp*_s of the mixtures. (Note *each* data point in supplementary Fig. S2 corresponds to *an* independently prepared sample.) We then established the *K*_*cp*_-composition relations for the mixtures, which were mostly linear (Supplementary Fig. S3) and could determine the *K*_*cp*_s of the PCs as pure substances. 16 independent samples were in average used to determine the *K*_*cp*_ of a PC, which is thus statistically sound and representative.

### Fusion Assay

Two populations of ULVs encapsulating either TbCl_3_ or DPA (fluorescent dyes) were prepared with extrusion^38^. A dried mixture of DOPE, PC and DOPA was prepared similarly as above, with the DOPA fixed at 4 mol% and *no* tetradecane added. (Adding DOPA was to introduce negative surface charges to facilitate the ULV formation; owing to the tiny, fixed amount and *C*_*0*,*DOPA*_ ≈ *C*_*0*,*DOPC*_ the DOPA contribution was negligible^15^,^39^). Buffer containing either (a) 2.5 mM TbCl_3_, 50 mM sodium citrate and 10 mM HEPES (pH = 7.4) or (b) 50 mM DPA and 10 mM HEPES (pH = 7.4) was used to suspend the lipid mixtures and the final lipid concentration was 5 mg/ml. Extrusion was carried out with Mini-Extruder system (Avanti) and a polycarbonate membrane at 40 °C. Unloaded dyes were removed with dialysis (3.5K MWCO, SnakeSkin dialysis tubing). The lipid concentrations were unchanged throughout the dialysis. Removal of unloaded dyes was confirmed by the lack of fluorescence from mixing the two ULV populations in the absence of PEG 8000. Small-angle X-ray scattering confirmed the ULV formation, rather than MLVs (Supplementary Fig. S4). The ULV size was measured with dynamic light scattering at 25 °C. Regardless of the composition, *all* our ULV samples had the average diameter of ~150 nm with narrow size distributions (Supplementary Fig. S5, upper panels).

Fusion was quantified with fluorescence from the DPA/TbCl_3_ complexes formed upon fusion of the LUVs. The assay was carried out by mixing the solutions of (a) DPA-loading LUVs, (b) TbCl_3_-loading LUVs, and (c) 50 wt% PEG 8000 and 1 mM EDTA, in the ratio of 100:100:800 μl. PEG 8000 initiated fusion by modifying the water chemical potential and hydration repulsion. The emission at 545 nm upon fusion was excited at 276 nm in a spectrofluorometer at 25 °C. The extent of fusion was determined as: Extent of fusion (%) = 
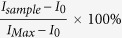
, where *I*_*sample*_ and *I*_*0*_ were the intensities of the sample at equilibrium and before fusion, respectively; and *I*_*Max*_ was the maximally possible intensity and obtained by adding 0.1% (v/v) Triton X-100, which disrupted the LUVs and released the dyes to maximize the number of the complexes formed. In measuring *I*_*sample*_, EDTA was present outside the LUVs to sequester leaked TbCl_3_ so that the detected emission was from fusion.

## Additional Information

**How to cite this article**: Fan, Z.-A. *et al.* Revisit the Correlation between the Elastic Mechanics and Fusion of Lipid Membranes. *Sci. Rep.* **6**, 31470; doi: 10.1038/srep31470 (2016).

## Supplementary Material

Supplementary Information

## Figures and Tables

**Figure 1:**
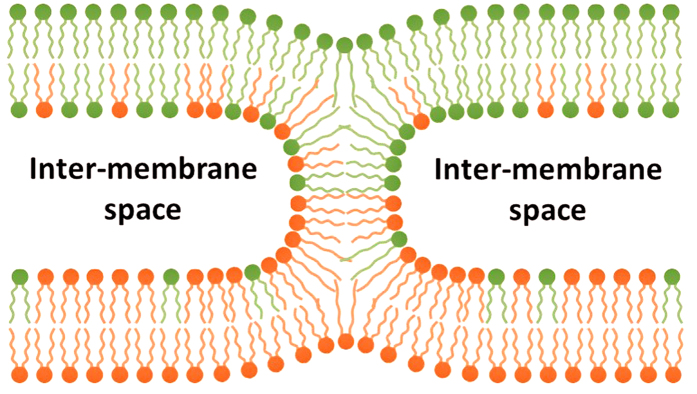
Hemifusion stalk. Color encodes the origins of the lipid molecules. In this fusion stage, the lipid molecules of the two *cis*-monolayers have intermixed while the *trans*-monolayers and the contents are still separated.

**Table 1:** *C*_*0*_ and *K*_*cp*_ of the studied lipids and LUVs.

		*C*_*0*_ (Å^−1^)						*K*_*cp*_ (×10^−19^ J)
Species		15 °C	20 °C	25 °C	30 °C	35 °C	40 °C	25 °C
DOPE		−0.0329 ± 0.0001	−0.0334 ± 0.0001	−0.0341 ± 0.0002	−0.0347 ± 0.0003	−0.035 ± 0.0002	−0.0366 ± 0.0001	0.57 ± 0.12
18:2 PE		−0.0345 ± 0.00004	−0.0350 ± 0.00005	−0.0356 ± 0.00002	−0.0361 ± 0.00005	−0.0365 ± 0.00007	−0.0379 ± 0.0002	0.33
18:3 PE		−0.0337 ± 0.00006	−0.0347 ± 0.00006	−0.0355 ± 0.00006	−0.0362 ± 0.00006	−0.0367 ± 0.00006	−0.0383 ± 0.00006	—
DOPC		−0.0094 ± 0.0005	−0.0102 ± 0.0007	−0.0106 ± 0.0008	−0.0111 ± 0.0010	−0.0116 ± 0.0009	−0.0120 ± 0.0011	—
18:2 PC		−0.0106 ± 0.0006	−0.0112 ± 0.0008	−0.0117 ± 0.0008	−0.0123 ± 0.0009	−0.0128 ± 0.0009	−0.0132 ± 0.0013	0.14
18:3 PC		−0.0086 ± 0.0007	−0.0095 ± 0.0014	−0.0099 ± 0.0015	−0.0104 ± 0.0017	−0.0109 ± 0.0019	−0.0113 ± 0.0021	0.14
DOPE/DOPC LUVs (mol% of DOPE)	**0**	—	—	−0.0106 ± 0.0008	—	—	—	0.40
	**25**	—	—	−0.0165 ± 0.0006	—	—	—	0.44
	**30**	—	—	−0.0177 ± 0.0006	—	—	—	0.45
	**40**	—	—	−0.02 ± 0.0005	—	–	—	0.47
	**50**	—	—	−0.0224 ± 0.0004	—	—	—	0.49
	**67**	—	—	−0.0264 ± 0.0003	—	—	—	0.51
Equimolar DOPE/PC LUVs (PC species)	**18:1**	—	—	−0.0224 ± 0.0008	—	—	—	0.49
	**18:2**	—	—	−0.0229	—	—	—	0.35
	**18:3**	—	—	−0.0220	—	—	—	0.36

**Table 2:** Calculated elastic energy densities of the LUVs.

		Elastic energy density (× 10^−3^ K_B_T/Å^2^)		
Species					*g*_*E*,*inner leaflet*_	*g*_*E*,*outer leaflet*_	*g*_*E*,*LUV*_
DOPE/DOPC LUVs (mol% of DOPE)	0	0.3	0.9	1.2
	25	1.0	2.0	3.0
	30	1.2	2.3	3.5
	40	1.7	2.9	4.6
	50	2.3	3.7	6.0
	67	3.5	5.2	8.7
Equimolar DOPE/PC LUVs (PC species)	18:1	2.3	3.7	6.0
	18:2	1.8	2.8	4.6
	18:3	1.6	2.6	4.2

**Table 3:** Elastic energy estimations for the monolayers of HD and FP in different compositions.

		Curvature energy term				Gaussian term			*G*_*E*_
Lipid composition		*K*_*cp*_ (×10^−19^ J)	*C* (Å)	*C*_*0*_ (Å)	*K*_*cp*_A(*C* − *C*_*0*_)^2^/2 (×10^−19^ J)	*K*_*G*_	*∫G*d*A*	*K*_*G*_*∫G*d*A* (×10^−19^ J)	(×10^−19^ J)
DOPE/DOPC	Negative *C*	0.49	−0.05 ~ −0.022	−0.022	1.67 ~ 0	−0.41	−4π	5.15	6.82 ~ 5.15
			−0.022 ~ 0		0 ~ 1.03				5.15 ~ 6.18
	Positive *C*		0 ~ 0.018		1.03 ~ 3.41				6.18 ~ 8.56
DOPE/18:2PC	Negative *C*	0.35	−0.05 ~ −0.022	−0.022	1.19 ~ 0	−0.29	−4π	3.64	4.84 ~ 3.64
			−0.022 ~ 0		0 ~ 0.74				3.64 ~ 4.38
	Positive *C*		0 ~ 0.018		0.74 ~ 2.44				4.38 ~ 6.08
