# Supplementary material for: Revisit the Correlation between the Elastic Mechanics and Fusion of Lipid Membranes
Source: Sci Rep. 2016 Aug 18;6:31470. doi: 10.1038/srep31470 (PMC4989284; doi:10.1038/srep31470)
Supplement: Supplementary Information [file srep31470-s1.pdf]

Supplementary Information for "Revisit the Correlation between the Elastic Mechanics and Fusion of Lipid Membranes"

Zih-An Fan, Kuan-Yu Tsang and Yi-Fan Chen*

DETAILED EXPERIMENTAL PROCEDURE AND DATA PROCESSING

Materials

Lipids, dioleoylphosphatidylethanolamine [DOPE or 18:1(9Z) PE; cat. no. 850725C], 18:2(9Z, 12Z) PE (denoted as 18:2 PE hereafter; cat. no. 850755C), 18:3(9Z, 12Z, 15Z) PE (denoted as 18:3 PE hereafter; cat. no. 850795C), dioleoylphosphatidylcholine [DOPC or 18:1(9Z) PC; cat. no. 850375C], 18:2(9Z, 12Z) PC (denoted as 18:2 PC hereafter; cat. no. 850385C), 18:3(9Z, 12Z, 15Z) PC (denoted as 18:3 PC; cat. no. 850395C) and dioleoyl phosphatidic acid [DOPA or 18:1(9Z) PA; cat. no. 840875C] were purchased from Avanti (Alabaster, AL) and used as received; the chloroform solutions of the lipids were stored at -20 ˚C before the sample preparation. Chemicals, tetradecane (cat. no. 87140), 2-methylbutane (cat. no. M32631), polyethylene glycol 8000 (PEG 8000; cat. no. P2139), HEPES buffer (cat. no. H0887), ethylenediaminetetraacetic acid (EDTA; cat. no. E9884), terbium (III) chloride hexahydrate ($TbCl_3 \cdot 6H_2O$; cat. no. 212903), sodium citrate dihydrate (cat. no. W302600), 2,6-pyridinedicarboxylic acid (DPA; cat. no. P63808) and Triton® X-100 solution (cat. no. 93443), were purchased from Sigma-Aldrich (St. Louis, MO) and used as received.

C_0 Measurement

In preparing the lipid dispersion samples for the C_0 measurements, the lipid stock solutions or their mixtures in the desired molar fractions were flushed with a gentle flow of argon gas, followed by an overnight incubation under vacuum, to thoroughly evaporate chloroform. Tetradecane, along with 2-methylbutane, was added to the dried lipid films. The resulting solutions were vigorously vortexed to homogenize the lipids and tetradecane. 2-methylbutane was subsequently evaporated following a similar procedure as for removing chloroform, except that the vacuum incubation was carefully timed such that 2-methylbutane was removed before the escape of tetradecane. Excessive buffer solution containing 10 mM HEPES with pH ≈ 7.4 was used to suspend the dried mixtures of lipid and tetradecane. The

obtained dispersions were shuffled between two glass syringes equipped with Teflon pistons [the syringe set of the Mini-Extruder (cat. no. 610000) from Avanti] for > 100 runs to improve the homogenization. The homogenization process was finished with >10 runs of freeze-thaw cycles. Each of the prepared samples had *a* fixed lipid composition and contained 16 wt% tetradecane.

The C_0s of the lipids were extracted from the X-ray diffraction data. The X-ray diffraction experiments were carried out with Cu $K\alpha$ and synchrotron radiations. The Cu $K\alpha$ radiation was generated from a rotating anode X-ray generator (NANO-Viewer, Rigaku, Japan) operating at 40 kV and 30 mA. The diffraction images were collected with a 2-D pixel detector (Pilatus 100K, Dectris, Switzerland). The synchrotron radiation was delivered by the beamlines of BL13A1 and BL23A1 at National Synchrotron Radiation Center in Hsinchu, Taiwan. The diffraction data generated with this X-ray source were collected with a Mar165 CCD 2-D detector and a Pilatus 1MF 2-D pixel detector at BL13A and BL23A1, respectively.

The data collection and processing schemes are detailed in Chen et. al. (2015). Briefly, a "homogeneity test", in which the X-ray beam was illuminated at different parts of a sample, was carried out first to examine the homogeneity of a sample; only the samples with the sole presence of the H_{II} phase and with no variations in the overall diffraction pattern and the positions of the diffraction peaks could proceed to the next stage of the data collection. The main X-ray data were collected at temperatures from 15 ˚C to 40 ˚C with a 5 ˚C interval. The collected data were reduced, azimuthally integrated and then background-subtracted. The thereby obtained 1-D diffraction profiles (*i.e.*, the plots of diffraction intensity, I_q, against $\vec{q}$, where $\vec{q} = 4\pi \sin\theta / \lambda$ with 2θ being the diffraction angle and λ the X-ray wavelength) were then used to reconstruct the electron density profiles of the H_{II} structures by exploiting the expression for the electron density, ρ_e, of a centrosymmetric unit cell (Harper et. al., 2001),

$$\rho_e(\vec{r}) = \rho_{avg} + \sum_q A_q \cos(\vec{q} \cdot \vec{r}),$$

where $\vec{r}$ is the position vector within the unit cell; ρ_{avg} is the average electron density; and A_q is related to I_q through,

$$A_q^2 \propto \frac{I_q sin\theta}{m},$$

with $\sin\theta \approx \theta$ (2θ is the diffraction angle) being the Lorentz correction and m the multiplicity factor. The phases of the diffraction peaks (*i.e.*, the signs of the A_qs) were "+" for the (1,0) peak; "-" for (1,1); "-" for (2,0); "+" for (2,1); "+" for (3,0); "+" for (2,2); and "+" for (3,1). The azimuthally averaged distance between the axis of the H_{II} water core and the electron dense region, where the phosphorous moieties and presumably the water-lipid interface were located, in a reconstructed electron density profile was defined as the radius of the water core, R_w. Following the method developed in Chen et. al. (2015), R_w could be used to calculate the radial distance, R_p, between the axis of the water core and the *pivotal plane* (pivotal plane is a dividing surface within the lipid monolayer of the H_{II} phase and has an unchanged cross-sectional area upon isothermal bending).

For the widely adopted C_0 determination method (e.g., Kirk and Gruner, 1985; Rand et. al., 1990) used in this present study, the H_{II} structure has to be solely formed by the lipids of interest under a constraint-free condition. A constraint-free condition can be achieved by adding free hydrocarbons (tetradecane in this study) and excess water to the lipid suspensions, to relieve the packing frustration among the hydrocarbon tails (Gruner, 1985) of the lipids and the hydration repulsion (Rand and Parsegian, 1989), respectively. Under this condition, the structural dimension of the H_{II} phase is expected to be mostly, if not exclusively, dictated by,

$$g_E = \frac{1}{2}K_{cp}(C - C_0)^2 + K_G G$$

where K_{cp} is the monolayer bending modulus, C the total curvature, C_0 the monolayer spontaneous curvature, K_G the Gaussian modulus and G the Gaussian curvature. By definition, the H_{II} phase has $G = 0$. Hence, the thermodynamically stable structure of the H_{II} phase shall have a total curvature of $C = C_0$ to minimize the g_E. The cylindrical geometry of the H_{II} phase entails C being equal to $1/R_p$, which could be derived from the experimentally determined R_w. Given the equivalence of C and C_0, C_0 was therefore obtained.

For the lipid species (*e.g.*, the PCs investigated in this study) favoring the lamellar phase, an experimental scheme detailed in Chen et. al. (2015) and elsewhere (e.g., Boulgaropoulos et. al., 2012) was employed. In this scheme, a (guest) lamellar-preferring lipid species is mixed with a (host) H_{II}-preferring one (*e.g.*, DOPE). A series of such binary lipid mixtures are prepared, with the molar fraction varied considerably but still carefully controlled such that the host lipid species dominates the composition and the H_{II} phase is the

sole stable phase in the presence of excess water and free hydrocarbons. The C_0 of the binary mixture, which is the molar-weighted average of the C_0s of the guest and host lipids and can then be measured with the method depicted above, displays a linear correlation with the molar fraction of the guest species (Fig. S1). By extrapolating this linear correlation to 100 mol% of the guest species, one can determine the C_0 of a lamellar-preferring lipid. Note that one single sample, with *a single* fixed lipid composition, could only contribute to one data point in Fig. S1, and at least three samples with the same lipid composition were prepared for each given composition to confirm the reproducibility and estimate the uncertainty.

K_{cp} Measurement

The protocols associated with the sample preparation, data collection and reconstruction of electron density profiles for the K_{cp} measurements were all identical to those for the C_0 measurements, except that for the K_{cp} measurements the buffer solutions also contained the water-soluble polymer, PEG 8000, and the X-ray diffraction data were taken only at 25 °C. Again, the lipid composition of a sample was carefully controlled such that the H_{II} phase was the sole stable phase in the presence of excess water and free hydrocarbons and the structural dimension was dictated by Equation 1.

Given the Flory radius of ~8 nm, the PEG molecules could not enter the H_{II} water core (<4 nm). Therefore, the polymer was concentrated in the bulk solution and applied an osmotic stress on the H_{II} structure; the magnitude of the osmotic stress depended on the PEG concentration (Stanley and Strey, 2003). The consequent structural deformation of the H_{II} phase was correlated with the osmotic stress through (Fuller et. al., 2003),

$$\Pi R_p^2 = 2K_{cp}\left(\frac{1}{R_p} - \frac{1}{R_{0p}}\right)$$

where Π is the applied osmotic stress and $R_{0p} = 1/C_0$. Once the electron density profiles were reconstructed for the H_{II} phase under different osmotic stresses, the K_{cp} of a H_{II}-forming lipid was determined through a ΠR_p^2-versus-$1/R_p$ plot, where the line slope was $2K_{cp}$.

To measure the K_{cp}s of the lamellar-preferring lipids, a new experimental scheme was developed. Similar to the case of the C_0 determination, a series of binary lipid mixtures (containing the H_{II}-forming DOPE and one of the lamellar-forming PCs) of varying molar fractions were prepared and placed under different osmotic stresses in the presence of the excessive buffer solution and 16 wt% tetradecane. The molar fractions of the binary mixtures

were carefully controlled such that the H_{II} phase was the sole stable structure for the mixtures and the K_{cp}s could be measured in a fashion similar to that for pure H_{II}-forming lipids. Fig. S2 presents part of our experimental data collected in this way. The repeat distance of the H_{II} unit cell shrinks with intensifying the osmotic stress and swells with increasing the molar fraction of 18:3 PC (Fig. S2a). Based on the datasets of this sort and the corresponding electron density profiles (reconstructed as described above), we were able to construct the ΠR_p^2-versus-$1/R_p$ plots for the DOPE/18:3 PC, as well as DOPE/18:2 PC, mixtures of various molar fractions (Fig. S2b). (It must be emphasized that *each* data point in Fig. S2a and S2b corresponds to *one* independently prepared sample.) The K_{cp} of one of the lipid mixtures could then be determined from the line slope of the plot. Based on a series of K_{cp} measurements, we established the correlations between the K_{cp} and lipid molar fraction for the binary mixtures composed of DOPE and 18:3 PC or DOPE and 18:2 PC. The correlations for both DOPE/18:3 PC and DOPE/18:2 PC are mostly linear (Fig. S3). (Note again that each data point in Fig. S3 was derived from a series of data points fitted with a dashed line in Fig. S2b and therefore from a collection of samples.) This suggests that we may be able to extract the K_{cp}s of the lamellar-forming PCs as pure substances by exploiting these linear correlations: One can extrapolate the linear correlations to 100 mol% of the guest PCs and thereby determine the K_{cp}s.

It has to be noted that the linearity between the K_{cp} and molar fraction is not unique to the lipid species studied here. Similar phenomena were also reported by Mitkova et. al. (2014), Genova et. al. (2014) and Chen and Rand (1997). This realization may suggest a wider-than-expected applicability of the method developed here. Moreover, 16 samples were in average used to determine the K_{cp}s of 18:2 PC or 18:3 PC. The K_{cp}s were therefore obtained from a large collection of samples, rather than from a small sample set. Thus, the K_{cp}s determined here are statistically sound and representative.

Fusion Assay

The samples used in the fusion assay were two populations of ULVs encapsulating $TbCl_3$ or DPA (both were fluorescent dyes), respectively. Preparation of the ULVs followed the conventional extrusion method (*e.g.*, reference). Briefly, a dried lipid mixture composed of DOPE, one of the three PC species and DOPA was prepared in the same manner as described above (except that *no* free hydrocarbon was added), with the molar fraction of DOPA fixed at

4 mol% and those of DOPE and PC varied. The buffer solution containing either (1) 2.5 mM $TbCl_3$, 50 mM sodium citrate and 10 mM HEPES (pH = 7.4) or (2) 50 mM DPA and 10 mM HEPES (pH =7.4) was used to suspend the lipid mixtures, with the final lipid concentration fixed in 5 mg/ml. The suspensions were subsequently homogenized with 5 freeze-thaw cycles and vigorous vortex. The extrusion was carried out by pressing the homogenized suspensions through the pore of a polycarbonate membrane placed in between two glass syringes of the Mini-Extruder system (cat. no. 610000, Avanti) at 40 ˚C. Three polycarbonate membranes with different orifice sizes (*i.e.*, 1 μm, 800 nm and 400 nm) were used in succession (from the larger to smaller pores). For each of the orifice sizes, the lipid mixtures were cycled between the two syringes for 31 runs. The unloaded fluorescent dyes ($TbCl_3$ or DPA) were removed by dialysis. This was carried out with a dialysis membrane tubing (3.5K MWCO, SnakeSkin dialysis tubing, Cat. no. 88242, Thermo Scientific, Waltham, MA). The lipid concentrations of the samples were unchanged throughout the dialysis. Complete removal of unloaded dyes was confirmed by measuring the fluorescence emitted from mixing the two populations of ULVs in the absence of PEG 8000 (the detail of the fluorescence measurements is described below). The formation of ULVs, rather than multilamellar vesicles (MLVs), in the samples was confirmed with small-angle X-ray scattering (Fig. S4). The sizes of the dye-encapsulating ULVs were measured with dynamic light scattering (Nanoparticle Analyzer, SZ-100, Horiba, Kyoto, Japan) at 25 ˚C. A great deal of efforts had been dedicated to ascertaining that *all* the prepared ULV samples, regardless of their lipid compositions, had the average diameter of ~150 nm with very narrow size distributions (Fig. S5, upper panels). This size consistency did not only further confirmed the formation of ULVs in all the samples but also dissipated the concern that size variations of the ULVs might have contributed to the changes in fusion of the ULVs. The diameter of ~150 nm classifies the ULVs to the category of large unilamellar vesicles (LUVs).

Fusion of the LUVs was quantified with the fluorescence intensities emitted by the DPA/$TbCl_3$ complexes formed upon fusion of the two LUV populations. This fusion assay were carried out by mixing (1) the solution of DPA-encapsulating LUVs, (2) the solution of $TbCl_3$-encapsulating LUVs, and (3) the solution of 50 wt% PEG 8000 and 1 mM EDTA, in the volume ratio of 100:100:800 μl. Fusion between the LUVs was initiated by PEG 8000, because its presence changed the water chemical potential and thus reduced the hydration repulsion (see Introduction in the main text) between the LUVs. The fluorescence emitted upon fusion was measured with a spectrofluorometer (FP-6500, Jasco, Easton, MD) at 25 ˚C. The DPA/$TbCl_3$ complexes were excited with light of 276 nm, and the fluorescent emissions

at 545 nm were recorded. The extent of fusion, presumably proportional to the fluorescence intensity, was determined against a reference through the equation,

$$\text{Extent of fusion (\%)} = \frac{I_{sample} - I_0}{I_{Max} - I_0},$$

where I_{sample}, I_0 and I_{Max} were the fluorescence intensities of the sample at equilibrium and before fusion, and maximum fluorescence intensity of the sample, respectively; I_{Max} presumably corresponded to the maximally possible extent of fusion and was obtained by adding to the samples 0.1% (v/v) Triton (a detergent that could disrupt the vesicular structure and release all of the content) to maximize the amounts of the complexes formed by DPA and $TbCl_3$, as well as the fluorescence intensity emitted from the samples. It is noted that in measuring I_{sample}, EDTA was present outside the LUVs to capture the $TbCl_3$ leaked from the LUVs so that the detected fluorescence intensity was emitted from fusion rather than from the leaked dyes.

SIZE MEASUREMENT FOR LIPOSOMES PRIOR TO AND FOLLOWING THE PEG 8000 ADDITION

Dynamic light scattering is carried out at 25 ˚C to examine the variations in the sizes of the DOPE/DOPC and DOPE/18:2 PC LUVs prior to and following the addition of PEG 8000. Before PEG 8000 is added, the two LUV species are both in the diameter of ~150 nm, as indicated above. Following the PEG 8000 addition, the size of the DOPE/DOPC LUVs expands considerably to the diameter of ~600 nm, consistent with the result of the fusion assay, where the content mixing and thus the occurrence of fusion are observed (Fig. S5a). Interestingly, the DOPE/18:2 PC LUVs also increases their diameter to >600 nm after the PEG 8000 addition, even though the fusion assay reveals no content mixing and thus no completion of the fusion process for the LUV species (Fig. S5b). The two observations indicate that the DOPE/18:2 PC LUVs can still approach one another but encounters difficulties in completing or even initiating the fusion process.

Reference

Boulgaropoulos B, Rappolt M, Sartori B, Amenitsch H, Pabst G (2012) Lipid sorting by ceramide and the consequences for membrane proteins. *Biophys J* 102(9):2031-2038.

Chen YF, Tsang KY, Chang WF, Fan ZA (2015) Differential dependencies on [Ca2+] and temperature of the monolayer spontaneous curvatures of DOPE, DOPA and cardiolipin: effects of modulating the strength of the inter-headgroup repulsion. *Soft Matter* 11(20):4041-4053.

Chen Z, Rand RP (1997) The influence of cholesterol on phospholipid membrane curvature and bending elasticity. *Biophys J* 73(1):267-276.

Genova J, Bivas I, Marinov R (2014) Cholesterol influence on the bending elasticity of lipid membranes. *J Colloids Surf A Physicochem Eng Aspects* 460:79-82.

Fuller N, Benatti CR, Rand RP (2003) Curvature and bending constants for phosphatidylserine-containing membranes. *Biophys J* 85(3):1667-1674.

Gruner SM (1985) Intrinsic curvature hypothesis for biomembrane lipid composition: a role for nonbilayer lipids. *Proc Natl Acad Sci USA* 82(11):3665-3669.

Harper PE, Mannock DA, Lewis RN, McElhaney RN, Gruner SM (2001) X-ray diffraction structures of some phosphatidylethanolamine lamellar and inverted hexagonal phases. *Biophys J* 81(5):2693-2706.

Kirk GL, Gruner SM (1985) Lyotropic effects of alkanes and headgroup composition on the lα -Hii lipid liquid crystal phase transition: hydrocarbon packing versus intrinsic curvature. *J Phys France* 46:761-769.

Lapinski MM, Castro-Forero A, Greiner AJ, Ofoli RY, Blanchard GJ (2007) Comparison of liposomes formed by sonication and extrusion: rotational and translational diffusion of an embedded chromophore. *Langmuir* 23(23):11677-11683.

Mitkova D, Marukovich N, Ermakov YA, Vitkova V (2014) Bending rigidity of phosphatidylserine-containing lipid bilayers inacidic aqueous solutions. *Colloids Surf A Physicochem Eng Aspects* 460:71-78.

Rand RP, Parsegian VA (1989) Hydration forces between phospholipid bilayers. *Biochim Biophys Acta* 988:351-376.

Rand RP, Fuller NL, Gruner SM, Parsegian VA (1990) Membrane curvature, lipid segregation, and structural transitions for phospholipids under dual-solvent stress. *Biochemistry* 29(1):76-87.

Stanley CB and Strey HH (2003) Measuring Osmotic Pressure of Poly(ethylene glycol) Solutions by Sedimentation Equilibrium Ultracentrifugation. *Macromolecules* 36(18):6888-6893.

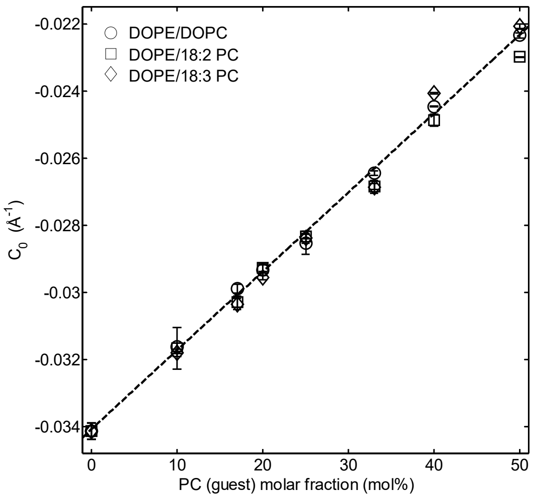

Fig. S1. C_0 as a function of PC fraction for the DOPE/DOPC, DOPE/18:2 PC and DOPE/18:3 PC mixtures at 25 ˚C.

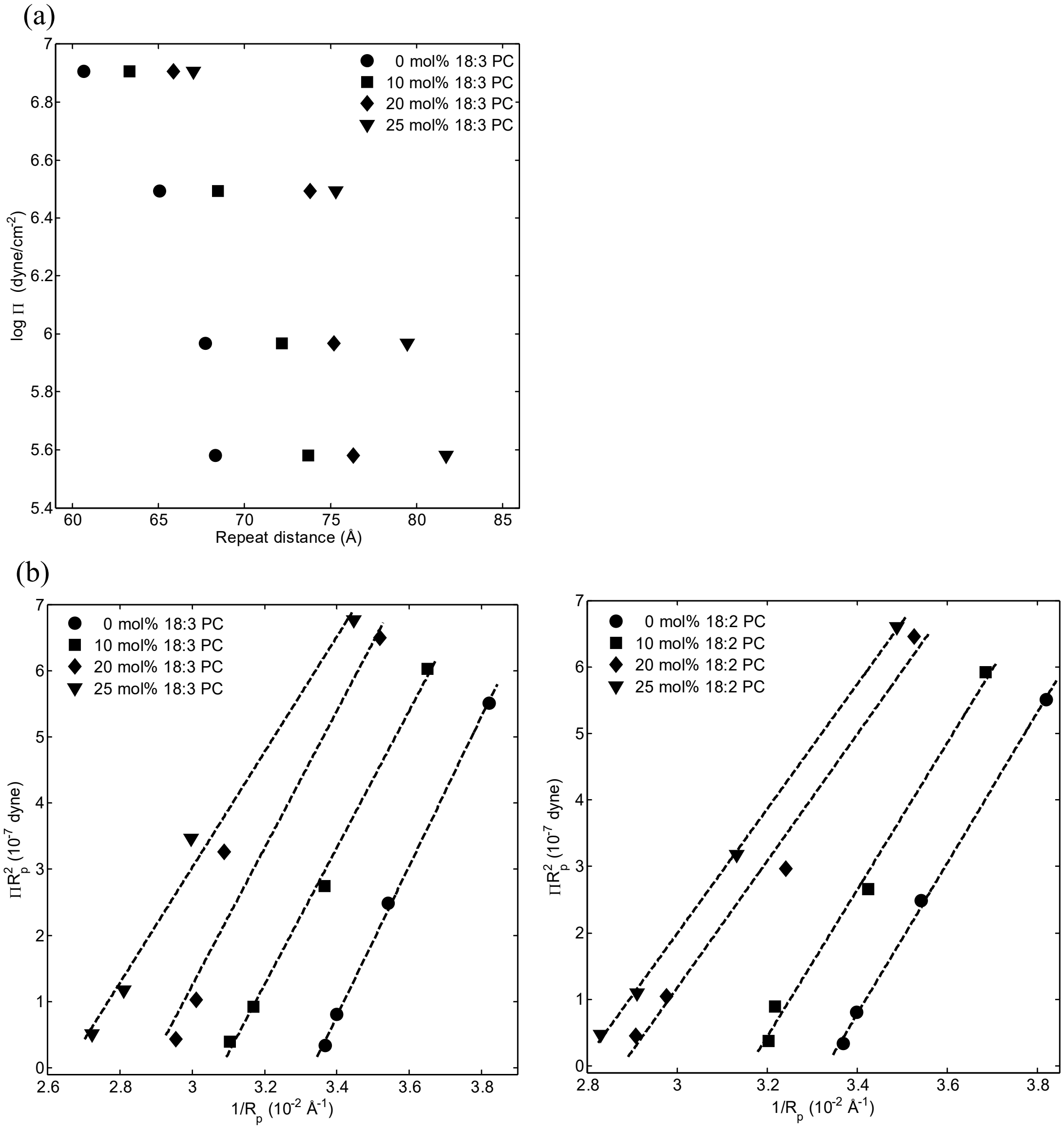

Fig. S2. The osmotic stress method is used to determine the K_{cp}s of lamellar-forming lipids. (a) Logarithmic osmotic stress (logΠ) as a function of repeat distance for the DOPE/18:3 PC mixtures in various molar fractions. (b) ΠR_p^2 against $1/R_p$ for the DOPE/18:3 PC (left) and DOPE/18:2 PC (right) mixtures in various molar fractions; the dashed lines are linear fits. A sample determines only one data point.

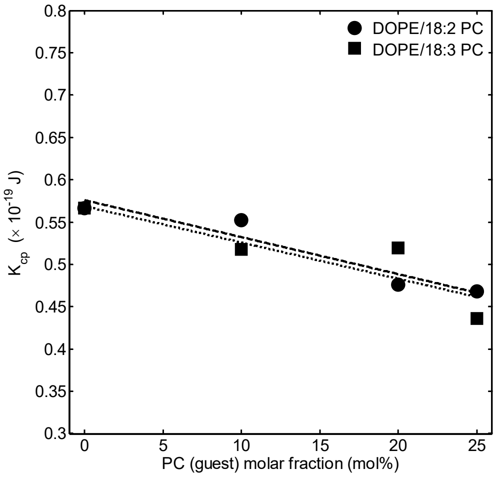

Fig. S3. K_{cp} as a function of PC fraction for the DOPE/18:3 PC and DOPE/18:2 PC mixtures. Good linearity is shown.

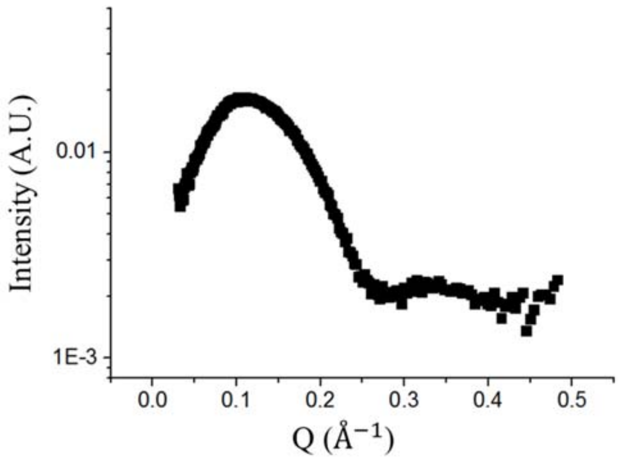

Fig. S4. X-ray scattering profile of the DOPE/DOPC LUVs.

(a)

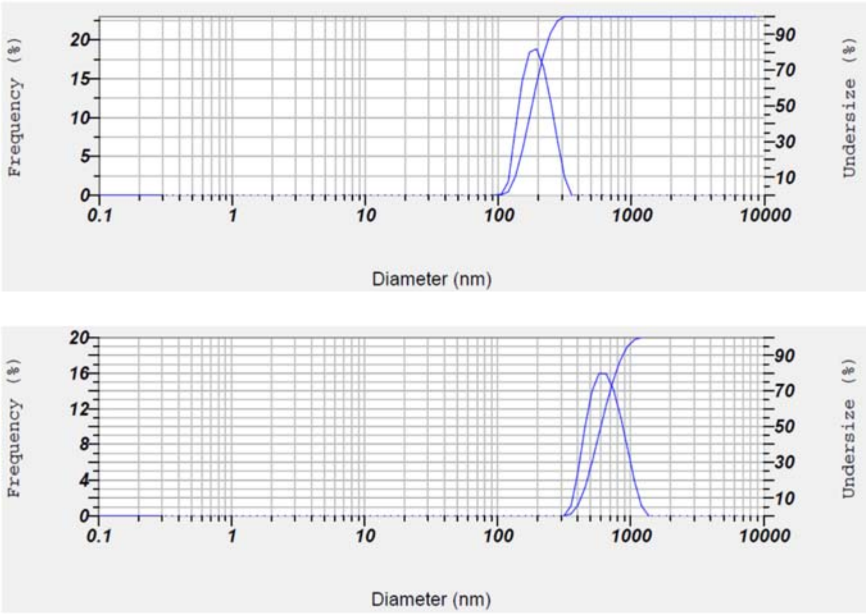

(b)

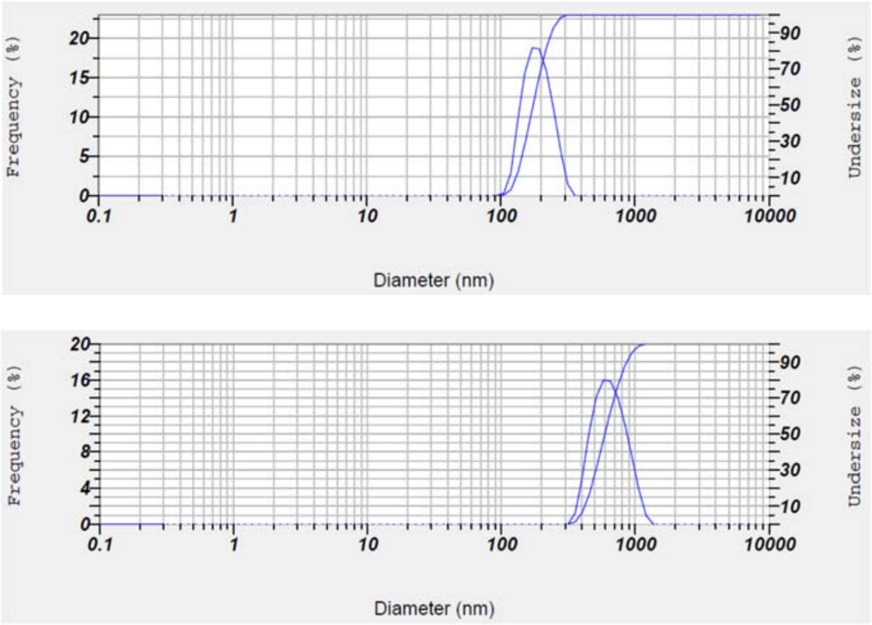

Fig. S5. The size distributions for (a) the DOPE/DOPC LUVs and (b) the DOPE/18:2 PC LUVs prior to (upper panel) and following (lower panel) the addition of PEG 8000.
